# Melatonin Promotes Improvement in Serum Lipid Levels and Liver Histopathology in Hyperlipidemic Rats

**DOI:** 10.1002/lipd.70056

**Published:** 2026-04-06

**Authors:** Ana Cláudia Carvalho de Sousa, Jaiurte Gomes Martins da Silva, Érique Ricardo Alves, Laís Caroline da Silva Santos, Bruno José do Nascimento, Marcelle Mariana Sales de França, Vanessa Bischoff Medina, Ismaela Maria Ferreira de Melo, Álvaro Aguiar Coelho Teixeira, Valéria Wanderley Teixeira

**Affiliations:** ^1^ Federal Rural University of Pernambuco (UFRPE) Recife Brazil; ^2^ Federal University of Alagoas (UFAL) Arapiraca Brazil; ^3^ Federal University of Mato Grosso do Sul (UFMS) Campo Grande Brazil

**Keywords:** antioxidant, biochemistry, cytokines, hepatic, hyperlipidemia

## Abstract

Hyperlipidemia or dyslipidemia is the term used for the increase in lipid levels in blood plasma, usually occurring due to a high‐fat diet associated with a sedentary lifestyle. The increase in lipid levels can cause fatty infiltration in the liver known as hepatic steatosis, which can lead to inflammation, fibrosis, and necrosis consecutively. Melatonin is the hormone primarily responsible for regulating the circadian cycle, but it has antioxidant and anti‐inflammatory properties and can also control lipid metabolism. Thus, the present research aimed to evaluate the effects of melatonin on the liver of hyperlipidemic rats. The animals were divided into control, tyloxapol, tyloxapol+melatonin. Hyperlipidemia was induced by applying tyloxapol at a dose of 400 mg/kg of body weight. Melatonin was administered simultaneously with tyloxapol in daily injections at a dose of 20 mg/kg. Sorological profiles for lipids, alkaline phosphatase, alanine aminotransferase, and aspartate aminotransferase, as well as liver histopathology and immunohistochemistry, were analyzed. Treatment with melatonin demonstrated an attenuating effect on the biochemical parameters of hyperlipidemic animals, reducing on average 80% the levels of triglycerides and very‐low‐density lipoprotein when compared to the tyloxapol group after 15 days of treatment, also showing a protective effect on the hepatic parenchyma that showed no changes. In addition, the administration of melatonin reduced the expression of proinflammatory cytokines and increased the expression of anti‐inflammatory ones. The present study showed that melatonin administration has a protective effect on induction factors and the development of nonalcoholic fatty liver disease in Wistar rats with hyperlipidemia.

## 1 Introduction

The hyperlipidemia or dyslipidemia is the term used for the increase in the levels of lipids (fats, cholesterol, and triglycerides [TGs]) in the blood plasma, which may be due to a genetic factor or acquired in many ways, such as diet, physical inactivity, and diabetes (Moneta 2022). A study carried out by Virani et al. (2021) revealed that in the United States about 12% of adults over the age of 20 years and 7% of children and adolescents, aged between 6 and 19 years, also have high cholesterol (Virani et al. 2021). In Brazil, the Brazilian Society of Cardiology estimates that 40% of adults have high cholesterol, whereas 38% of men and 42% of women have it above 200 mg/dL (Sposito et al. 2007). Individuals who have hyperlipidemia may not only develop cardiovascular diseases but also diseases in other organs, such as the liver and kidneys (Zámbó et al. 2013).

Increased blood lipid levels can cause fat infiltration into the liver, where excess TG and cholesteryl esters are stored as droplets in the cytoplasm of hepatocytes resulting in nonalcoholic fatty liver disease (NAFLD), which occurs in the absence of consumption of alcohol and whose pathogenesis is still the subject of studies (Assay et al. 2000; Cao et al. 2017; Sweet et al. 2017). NAFLD can change the metabolic status of the liver, being of great importance to evaluate the activity of transaminase enzymes, which act in the metabolism of amino acids, with the release of these enzymes in the plasma, especially ALT (alanine aminotransferase), when there is damage to the liver (Leite et al. 2016).

NAFLD begins with the accumulation of fat in the liver, whose stages involve the process of inflammation, fibrosis and necrosis, consecutively (Browning and Horton 2004; Ma et al. 2008; Das and Balakrishnan 2011). Studies show that cytokines are important mediators of hepatosteatosis, as they can cause severe systemic and local changes, such as lobular inflammation and ballooning of hepatocytes, the most important cytokines being TNF‐α (tumor necrosis factor α), IL‐1 (interleukin 1), IL‐6 (interleukin 6), and interferon (Das and Balakrishnan 2011; Barr et al. 2016).

Melatonin is the hormone produced by the pineal gland and is responsible for providing information about the circadian cycle, and its production is regulated by light (Imenshahidi et al. 2020). In addition, it also has antioxidant and anti‐inflammatory properties and can also control lipid metabolism (Imenshahidi et al. 2020). These properties protect cells and tissues from damage caused by free radicals, being able to inhibit proinflammatory cytokines such as TNF‐α, IL‐1 and IL‐6, and consequently reduce damage to hepatocytes, thus decreasing the chances of cell death (El‐Sokkary et al. 2010; Shajari et al. 2015; Esteban‐Zubero et al. 2016; Reiter et al. 2016).

Based on the data presented and the lack of literature on the role of melatonin on cytokines and hepatic histophysiology during hyperlipidemia, the present study aimed to analyze the possible effects of melatonin on histopathological, immunohistochemical, and biochemical parameters of the liver of hyperlipidemic animals.

## 2 Methods

### 2.1 Animals

Thirty albino Wistar rats, 60 days old and weighing approximately 250 ± 30 g, from the Bioterium of the Department of Animal Morphology and Physiology of the Federal Rural University of Pernambuco (UFRPE), were kept in an environment with temperature (22°C ± 1°C) and photoperiod (12 h light and 12 h dark) controlled and in ad libitum feeding regime. All animal care and study protocols were approved by the Ethics Committee of UFRPE.

### 2.2 Experimental Groups

The animals were randomly divided into three experimental groups. Control group: animals without hyperlipidemia induction; tyloxapol group: hyperlipidemic animals; tyloxapol + melatonin group: hyperlipidemic animals treated with Melatonin.

### 2.3 Induction of Hyperlipidemia

This was performed by applying Triton WR 1339, also known as tyloxapol (Sigma‐Aldrich), at a dose of 400 mg/kg of body weight, dissolved in 0.9% NaCl, by intraperitoneal injection, on alternate days for 15 days (Salamaa et al. 2012). Control animals were injected with the same vehicle.

### 2.4 Melatonin Treatment

Melatonin, *N*‐acetyl‐5‐methoxytryptamine (M5250‐5G, Sigma Chemical Co., St. Louis, USA) was administered in daily injections for 15 days, intraperitoneally from 6:00 p.m. to 7:00 p.m., at 20 mg/kg; this was dissolved in 0.2 mL of ethanol and diluted in 0.9 mL of 0.9% NaCl. Control animals were injected with the same hormone vehicle.

### 2.5 Organ Collection (Liver)

For liver collection and animal euthanasia, the rats were anesthetized with ketamine hydrochloride (80 mg/kg) and xylazine (6.0 mg/kg) intramuscularly, associated with thiopental (100 mg/kg), intraperitoneally. Liver fragments were fixed in buffered formalin, remaining there for 48 h. After these procedures, they were dehydrated in increasing concentrations of ethyl alcohol, diaphanized with xylene, impregnated, and embedded in paraffin. The paraffin blocks were cut in a Minot microtome and submitted to the staining technique by hematoxylin and eosin (HE).

### 2.6 Liver Morphometry

The morphometric study was performed using stereological methods, according to the methodology described by Engelman et al. (2001) analyzing the proportion between the non‐lobular and lobular parenchyma of the liver of the experimental groups, using a grid with 100 test points, placed on the sections of the histological preparations stained by HE. The count was performed on three slides, so that, 10 fields were counted using the ×40 objective, making a total of 3000 points per group.

A semiquantitative assessment of the scores of liver changes proposed by the Pathology Committee of the NASH (nonalcoholic steatohepatitis) Clinical Research Network (Kleiner et al. 2005) was performed, which are required to follow: steatosis (< 5% = 0; 5%–33% = 1; 33%–66% = 2; > 66% = 3); lobular inflammation (none = 0; 2 foci = 1; 2–4 foci = 2; > 4 foci = 3); and hepatocellular ballooning (none = 0; few = 1; prominent = 2). All features were scored blindly on three slides/group, evaluated five fields/slide. For this, the images were captured with a Sony video camera coupled to an Olympus BX50 microscope. All quantifications were performed in the image editor Gimp 2.8.

### 2.7 Biochemical Analysis

Blood collection was performed on the seventh day after the start of treatment, by lateral caudal puncture. On the day of euthanasia, blood was collected by cardiac puncture. The material was centrifuged and stored at −20°C until the day of biochemical analysis. The levels of HDL, LDL, VLDL, TG, and CT were analyzed using commercial kits from Labtest and Doles using a spectrophotometer. The levels of alkaline phosphatase and transaminases (aspartate aminotransferase [AST] and ALT) were analyzed using commercial kits from Bioclin using a spectrophotometer.

### 2.8 Immunohistochemical Analysis (IL‐6, TNF‐α, IL‐4, and IL‐10)

Antigenic recovery was performed using a citrate buffer solution (pH 6.0) at high temperature in the microwave for 5 min. Endogenous peroxidase was inhibited by a solution of hydrogen peroxide (3%) in methanol. The nonspecific antigen–antibody reaction was blocked by incubating the slides in PBS and 5% bovine serum albumin (BSA) for 1 h. All antibodies (Santa Cruz Biotechnology Inc., Santa Cruz, CA, USA) were diluted in PBS/1% BSA overnight. Subsequently, the slides were treated with the secondary antibody, histofine, for 30 min. The antigen–antibody reaction was observed through a brown precipitate after application of 3,3′‐diaminobenzidine for 4 min and counterstained with hematoxylin (Oberholzer et al. 1996; Lee et al. 2001).

### 2.9 Statistical Analysis

To compare the morphometric data, analysis of variance was performed; when significant, it was complemented by the Tukey and Kramer multiple comparisons tests (*p* < 0.05).

## 3 Results

### 3.1 Biochemical Analysis

The biochemical analyzes at 7 days of treatment revealed a significant increase in the serum levels of TGs, cholesterol, alkaline phosphatase, ALT, and AST in the animals of the tyloxapol group, in relation to the other experimental groups. Treatment with melatonin showed reduced values in the levels of these parameters, with a reduction of 47.44% for TGs and 93.61% and 86.92% for ALT and AST, respectively, but these parameters remain high in relation to the control. Regarding LDL and VLDL, there were no significant differences between the tyloxapol and tyloxapol + melatonin groups; however, both differed from the control. There were no differences in HDL levels between the experimental groups (Table 1).

**TABLE 1:** Mean ± standard deviation of the biochemical parameters in the animals of the experimental groups, at 7 days of treatment.

Groups	Control	Tyloxapol	Tyloxapol + melatonin	*p*
TG (UI/L)	61.32 ± 6.87c	5646.00 ± 264.91a	2968.02 ± 499.97b	0.0029
LDL (mg/dL)	18.02 ± 1.10b	74.71 ± 23.29a	106.45 ± 13.97a	0.0312
VLDL (mg/dL)	9.23 ± 1.15b	422.53 ± 2.61a	431.12 ± 8.10a	0.0001
HDL (mg/dL)	60.25 ± 1.75a	72.25 ± 9.93a	68.70 ± 5.03a	0.5387
TC (mg/dL)	87.50 ± 1.80c	544.57 ± 45.00a	348.82 ± 17.75b	0.0002
ALP (UI/L)	90.60 ± 0.25c	412.44 ± 17.79a	141.59 ± 18.14b	0.0001
ALT (UI/L)	10.82 ± 0.31c	434.36 ± 31.90a	27.77 ± 6.48b	0.0001
AST (UI/L)	59.88 ± 2.53c	606.27 ± 3.87a	79.31 ± 3.49b	0.0001

*Note:* Means followed by the same letters in the lines do not differ significantly from each other by the Tukey and Kramer multiples test (*p* < 0.05).

Abbreviation: ALP = alkaline phosphatase.

At 15 days of treatment, there was a significant increase in the serum levels of TGs, LDL, VLDL, and cholesterol in the animals of the tyloxapol group in relation to the other experimental groups. The hyperlipidemic group treated with melatonin showed reduced values in the levels of these parameters, with a reduction of 83.67% for TGs, 86.29% for VLDL, and 69.37% for total cholesterol, but these parameters remained high in relation to the control. Serum levels of alkaline phosphatase, ALT, and AST were elevated in relation to those observed in animals in the control and tyloxapol + melatonin groups, which did not differ from each other. There was a significant reduction in HDL levels in the animals of the tyloxapol group when compared to the other groups; however, the treatment with melatonin showed the highest levels (Table 2).

**TABLE 2:** Mean ± standard deviation of the biochemical parameters in the animals of the experimental groups, at 15 days of treatment.

Groups	Control	Tyloxapol	Tyloxapol + melatonin	*p*
TG (UI/L)	81.61 ± 24.40c	6662.87 ± 125.87a	1088.11 ± 331.34b	0.0158
LDL (mg/dL)	18.88 ± 2.46c	59.51 ± 16.27a	36.05 ± 7.050b	0.0114
VLDL (mg/dL)	8.87 ± 1.89c	424.00 ± 5.27a	58.15 ± 34.65b	0.0013
HDL (mg/dL)	56.40 ± 3.70b	44.05 ± 5.55c	99.10 ± 5.00a	0.0080
TC (mg/dL)	84.15 ± 0.65c	533.03 ± 5.65a	163.36 ± 8.15b	0.0049
ALP (UI/L)	90.66 ± 5.10b	432.82 ± 11.59a	98.84 ± 4.64b	0.0001
ALT (UI/L)	11.48 ± 1.19b	450.00 ± 20.38a	13.75 ± 1.57b	0.0001
AST (UI/L)	60.22 ± 2.55b	602.46 ± 1.98a	52.47 ± 7.32b	0.0001

*Note:* Means followed by the same letters in the lines do not differ significantly from each other by the Tukey and Kramer multiples test (*p* < 0.05).

Abbreviation: ALP = alkaline phosphatase.

### 3.2 Histopathological and Morphometric Analysis

**FIGURE 1:**
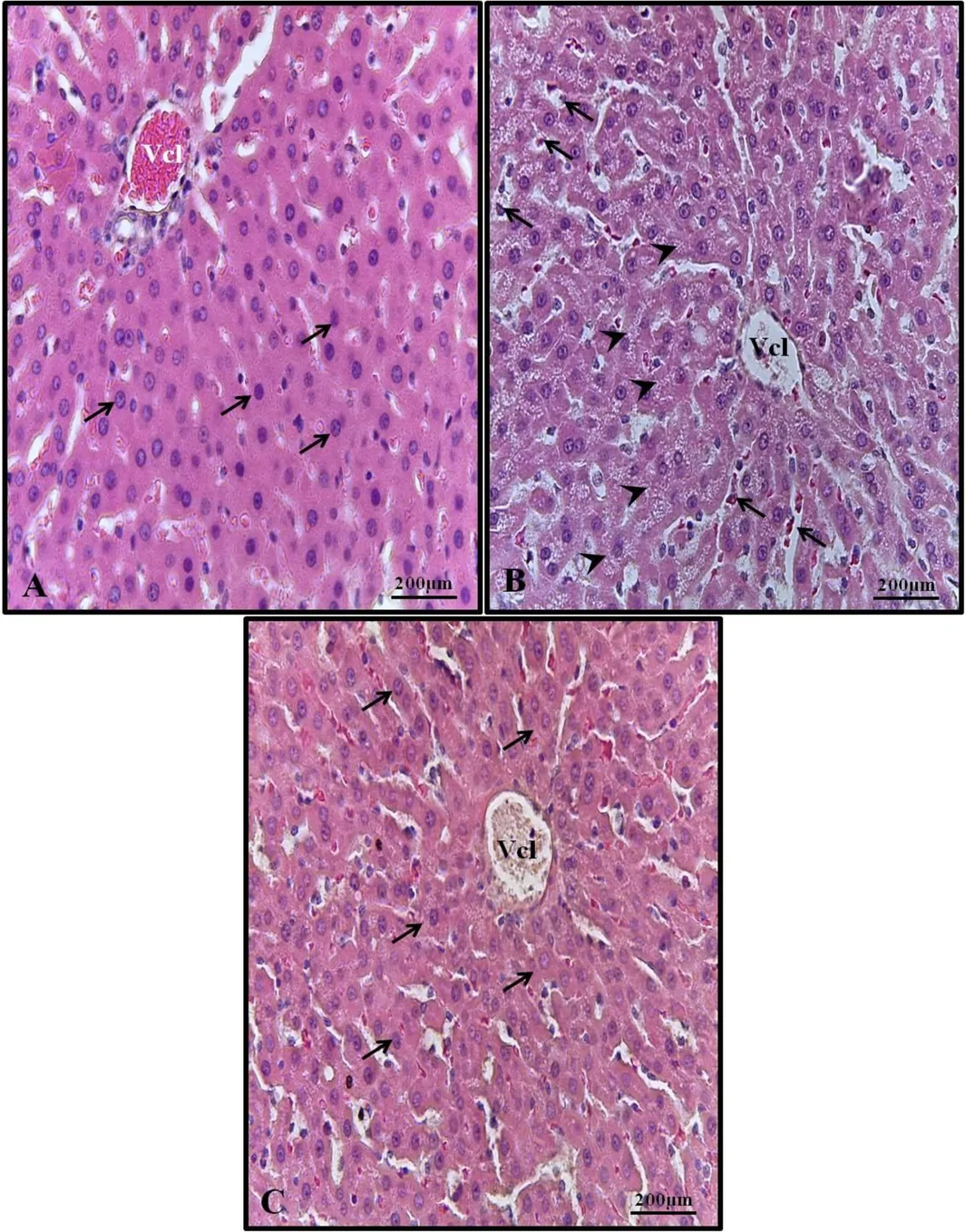
Photomicrograph of the liver of animals in the experimental groups. (A) Control; (B) tyloxapol; and (C) tyloxapol + melatonin. Check hepatocytes without steatosis (long arrows) in (A and C), and hepatocytes with steatosis (arrowheads) in (B), characterized by the presence of numerous cytoplasmic vacuoles, in addition to leukocyte infiltrate (short arrows). Vcl, centrilobular vein. HE, hematoxylin and eosin.

Histopathological analysis of the liver of animals in the control and tyloxapol + melatonin groups revealed unaltered liver parenchyma with organized hepatocyte cords surrounding the centrilobular vein, interspersed by sinusoid capillaries. However, in the livers of the rats in the hyperlipidemic group, steatosis and leukocyte infiltration were observed (Figure 1).

There was a reduction in the percentage of lobular parenchyma and an increase in non‐lobular parenchyma in the liver of animals in the tyloxapol group. Melatonin prevented this effect, presenting similar parameters about the control group (Table 3).

**TABLE 3:** Mean ± standard deviation of the percentage of lobular and non‐lobular parenchyma in the liver of females from the experimental groups.

Groups	Control	Tyloxapol	Tyloxapol + melatonin	*p*
Lobular parenchyma	85.76 ± 2.09a	73.12 ± 1.98b	82.33 ± 3.77a	0.0111
Non‐lobular parenchyma	14.24 ± 1.55b	26.88 ± 3.17a	17.67 ± 4.09b	0.0432

*Note:* Means followed by the same letters in the lines do not differ significantly from each other by the Tukey and Kramer multiples test (*p* < 0.05).

**FIGURE 2:**
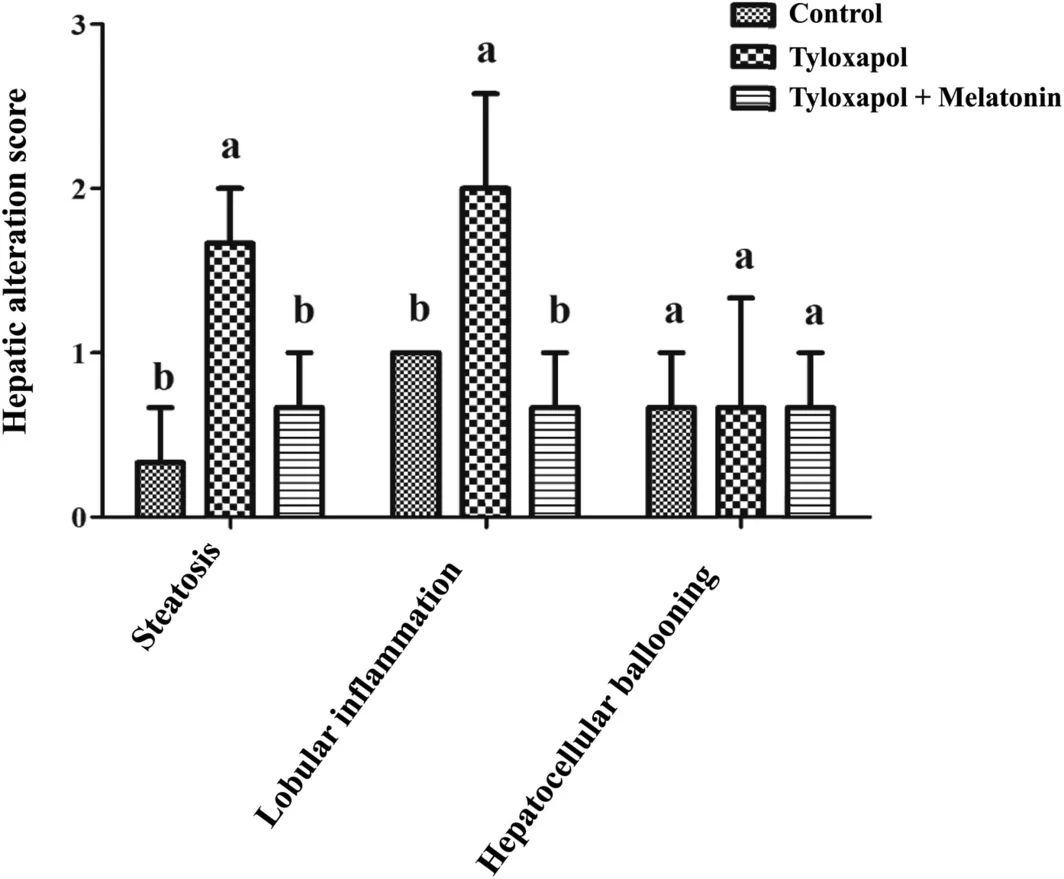
Graph of the score of liver changes in animals in the experimental groups. To verify a significant increase in steatosis and lobular inflammation in the tyloxapol group. Means followed by the same letter did not differ significantly from each other by the Tukey and Kramer test (*p* < 0.05).

In the semiquantitative evaluation of the scores of the hepatic alterations, it was revealed that the animals of the tyloxapol group had high scores of lobular inflammation and of hepatocytes with steatosis. Treatment with melatonin considerably reduced these effects, with scores similar to the control group. There were no significant differences in hepatocyte ballooning scores between the experimental groups (Figure 2).

### 3.3 Immunohistochemical Analysis (IL‐6, TNF‐α, IL‐4, and IL‐10)

**FIGURE 3:**
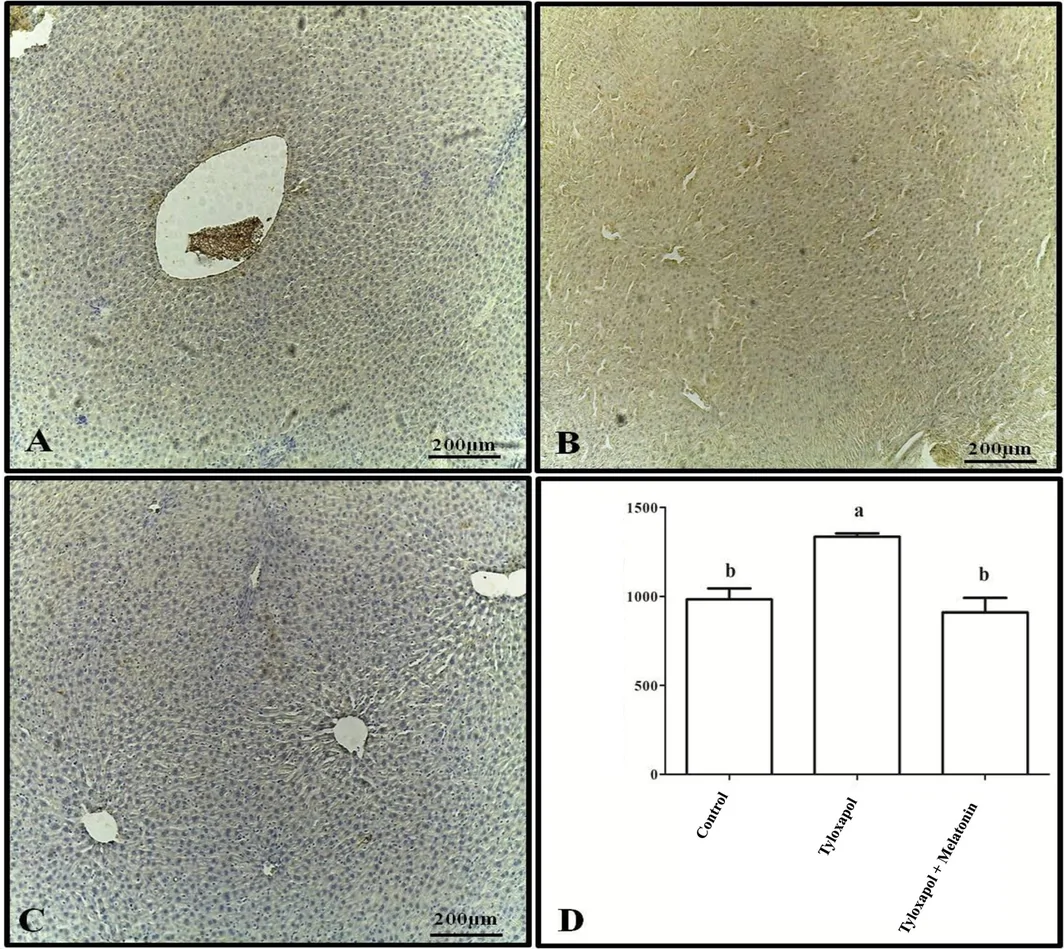
Immunohistochemistry for IL‐6 in the liver of animals in the experimental groups. (A) Control; (B) tyloxapol; and (C) tyloxapol + melatonin. Observe the stronger labeling in (B). (D) Quantification in pixels of the IL‐6 expression. Note elevation in the tyloxapol group. Means followed by the same letter do not differ significantly from each other in the Tukey and Kramer multiple comparisons test (*p* < 0.05).

**FIGURE 4:**
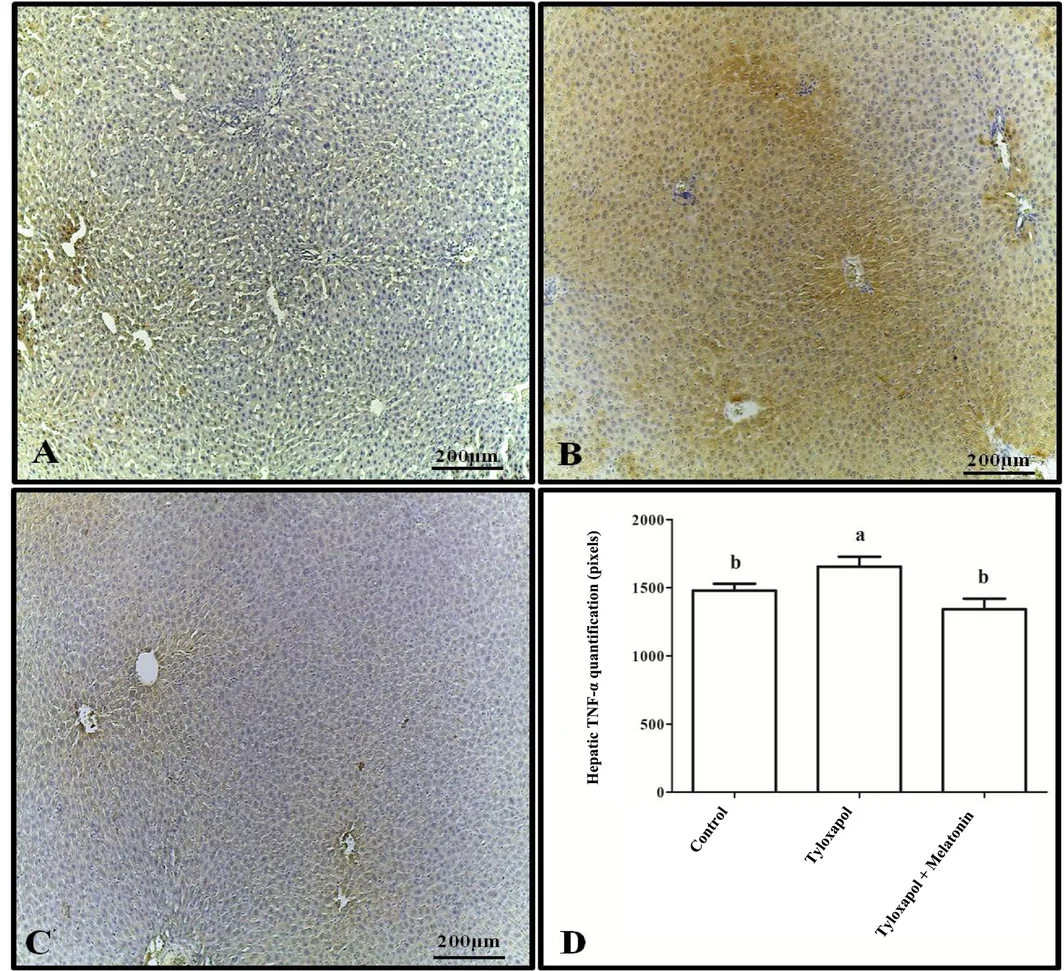
Immunohistochemistry for TNF‐α in the liver of animals in the experimental groups. (A) Control; (B) tyloxapol; and (C) tyloxapol + melatonin. Observe the stronger labeling in (B). (D) Quantification in pixels of the expression of TNF‐α. Note elevation in the tyloxapol group. Means followed by the same letter do not differ significantly from each other in the Tukey and Kramer multiple comparisons test (*p* < 0.05).

**FIGURE 5:**
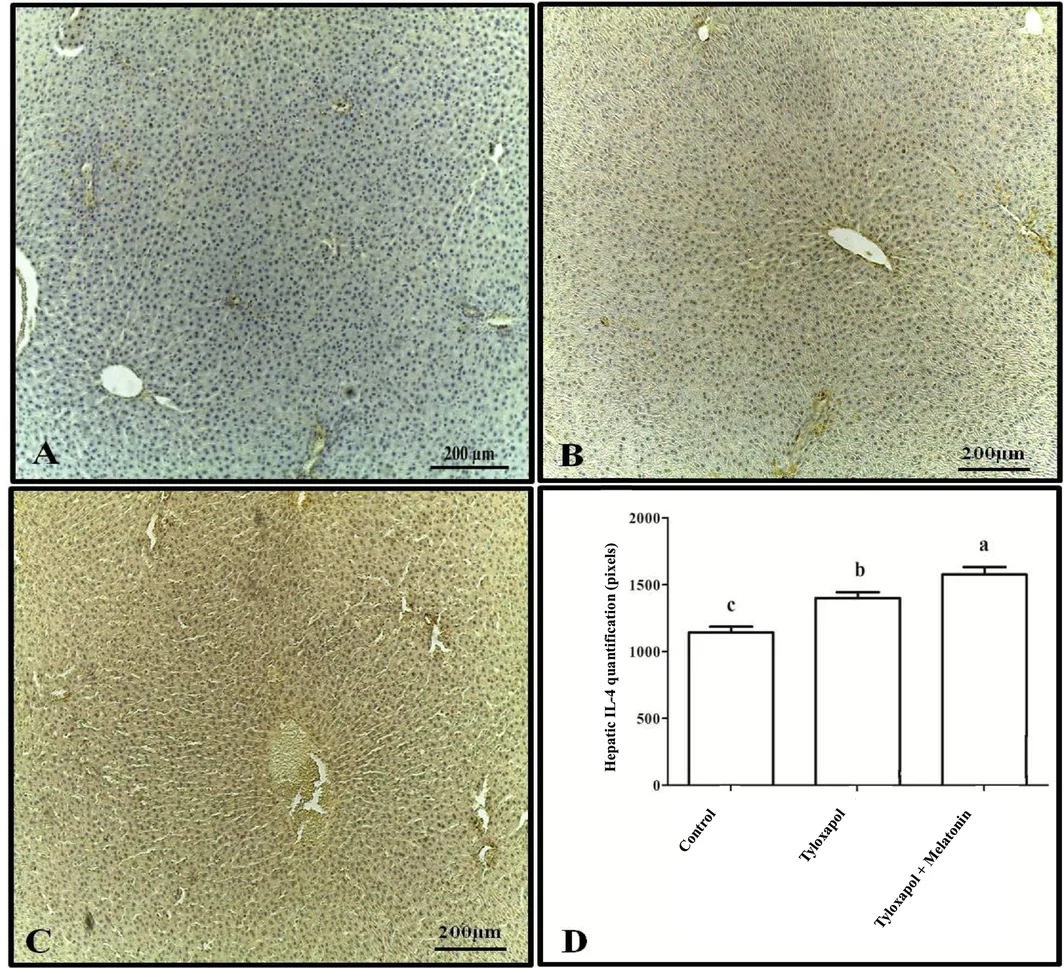
Immunohistochemistry for IL‐4 in the liver of animals in the experimental groups. (A) Control; (B) tyloxapol; and (C) tyloxapol + melatonin. Check moderate labeling in (B) and strong in (C). (D) Quantification in pixels of IL expression 4. Note an increase in the tyloxapol + melatonin group. Means followed by the same letter do not differ significantly from each other in the Tukey and Kramer multiple comparisons test (*p* < 0.05).

**FIGURE 6:**
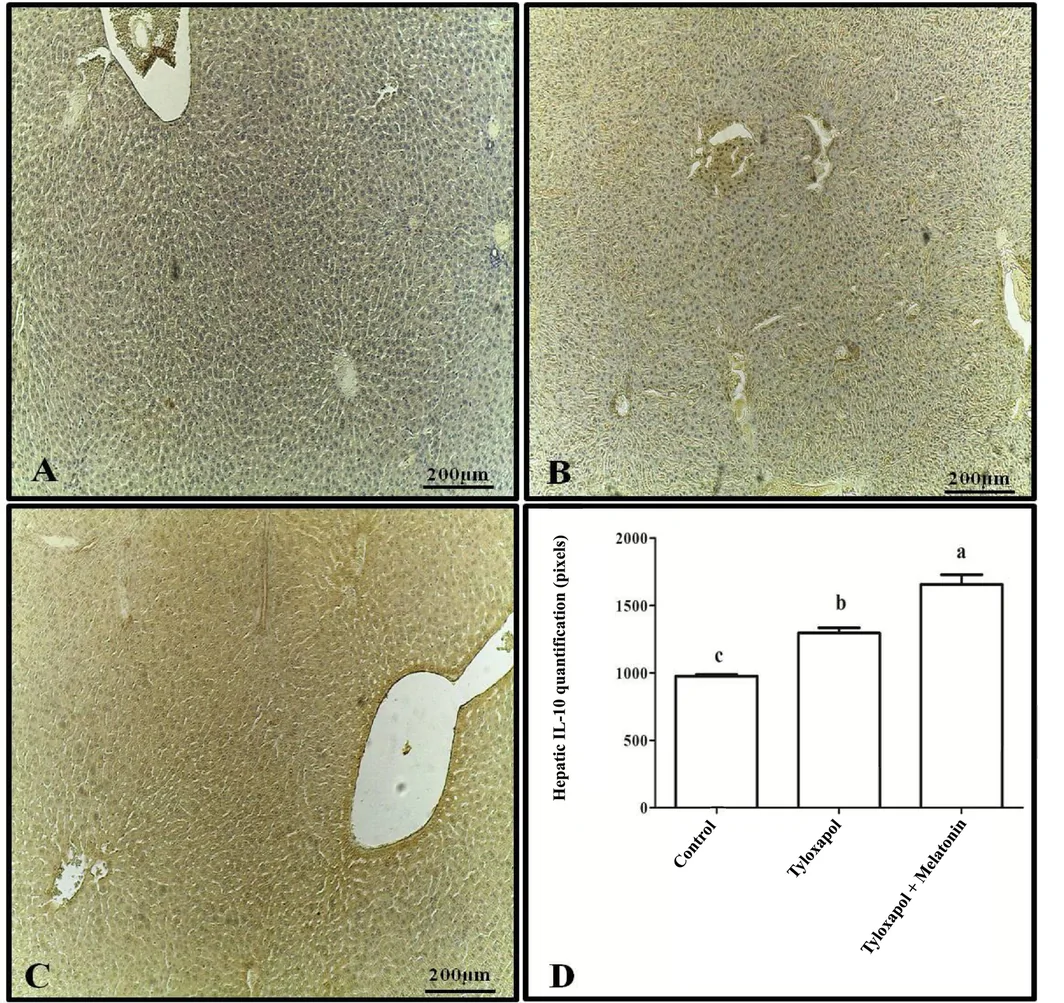
Immunohistochemistry for IL‐10 in the liver of animals in the experimental groups. (A) Control; (B) tyloxapol; and (C) tyloxapol + melatonin. Check moderate labeling in (B) and strong in (C). (D) Quantification in pixels of IL 10 expression. Note an increase in the tyloxapol + melatonin group. Means followed by the same letter do not differ significantly from each other in the Tukey and Kramer multiple comparisons test (*p* < 0.05).

The immunohistochemical analysis of proinflammatory cytokines showed a significant increase in the expression of IL‐6 and TNF‐α in the livers of the animals of the tyloxapol group when compared to the others (Figures 3 and 4). However, in relation to the anti‐inflammatory cytokines IL‐4 and IL‐10, there was moderate staining in the livers of the animals of the tyloxapol group and strong staining in the treatment with melatonin (Figures 5 and 6).

## 4 Discussion

The biochemical analysis demonstrated the effectiveness of tyloxapol in inducing hyperlipidemia, in which, on the seventh day of treatment, an increase in TG, LDL, VLDL, and TC levels was observed, with a significant increase in these parameters, accompanied by a reduction in HDL levels on the fifteenth day. The fifteenth day of treatment in this same group. These results are consistent with several studies, since tyloxapol is able to inhibit the action of the lipoprotein lipase enzyme, responsible for TG hydrolysis, and to increase the action of the hydroxymethylglutaryl‐CoA reductase enzyme, which is responsible for increased cholesterol synthesis in hepatic cells (Linarelli and Pott Jr. 2008). Additionally, the excess of circulating lipids induced by tyloxapol is associated with direct metabolic effects related to the activation of lipid‐sensitive inflammatory and metabolic pathways, such as the NLRP3 inflammasome, nuclear factor κ‐light‐chain‐enhancer of activated B cells (NF‐κB), and the lipogenic regulators SREBP‐1c and PPAR‐α, which contribute to the hepatic alterations observed (Kuo et al. 2020; Zhang et al. 2025).

The increase in serum lipid levels leads to fat accumulation in the liver, causing simple steatosis, also known as nonalcoholic fatty liver, which may progress to nonalcoholic hepatic steatosis (NASH), which can promote liver damage. This was observed in the group treated only with tyloxapol and was also evidenced by increased serum levels of alkaline phosphatase, ALT, also known as glutamic pyruvic transaminase, and AST, also called glutamic oxaloacetic transaminase (Younossi et al. 2018). The occurrence of NASH in this group can also be confirmed by histopathological and morphometric analyses, which showed high scores of lobular inflammation and steatotic hepatocytes in the liver of tyloxapol‐treated animals, in addition to a reduction in the percentage of lobular parenchyma and an increase in non‐lobular parenchyma in the liver, probably due to fibrosis, which is also one of the consequences of NASH (Younossi et al. 2018).

The pathogenesis of nonalcoholic hepatic steatosis develops through several mechanisms, one of which is lipotoxicity, which may cause hepatocyte apoptosis, promoting the release of inflammatory content after necrosis by immune cells present in the liver, such as leukocytes, macrophages, and Kupffer cells, with TNF‐α and IL‐6 being the inflammatory cytokines released by these cells (Tilg and Moschen 2010; Alkhouri et al. 2011; Malhi and Kaufman 2011). This lipotoxicity induced by tyloxapol treatment can be verified by immunohistochemistry, with increased immunohistochemical labeling for TNF‐α and IL‐6 and the presence of leukocyte infiltrates in histopathological analyses. In this context, the accumulation of fatty acids and lipotoxicity‐derived products may result in activation of Toll‐like receptor 4 (TLR4), culminating in NF‐κB activation, which leads to increased production of TNF‐α and IL‐6. In addition, lipotoxicity is associated with activation of the NLRP3 inflammasome and endoplasmic reticulum stress, mechanisms that intensify hepatic inflammation and hepatocellular damage (Lee et al. 2003; Rogero and Calder 2018).

Melatonin treatment demonstrated a protective effect on the biochemical parameters of hyperlipidemic animals, promoting an average reduction of approximately 80% in TG and VLDL levels after 15 days of treatment, although these parameters remained elevated compared to the control group. In addition, melatonin prevented fat accumulation in the liver, attenuating the development of hepatic steatosis and, consequently, lobular inflammation, which was corroborated by serum levels of alkaline phosphatase, ALT, and AST similar to those observed in the control group. This hepatoprotective effect is probably related to the ability of melatonin to inhibit cholesterol absorption and accumulation in hepatocytes, since it inhibits the action of the cholesterol acetyltransferase enzyme (acyl‐CoA), responsible for cholesterol esterification, thereby reducing both hepatic cholesterol accumulation and plasma cholesterol levels (Kreeman et al. 1998; Skottova et al. 2001; Hussain 2007). It is also noteworthy that acyl‐CoA plays a central role in several aspects of lipid metabolism, including lipoprotein and steroid hormone biosynthesis, as well as foam cell formation in atheroma (Chang et al. 2001). Additionally, melatonin may modulate the expression of genes involved in hepatic lipogenesis, contributing to reduced lipid accumulation and improvement of the metabolic profile observed in treated animals (Dang et al. 2025).

Chan and Tang (1995) demonstrated in their experiments that melatonin facilitates hepatic bile acid catabolism and, consequently, their elimination through feces, which would probably explain the increase in HDL observed in the melatonin‐treated group compared to the control group, since HDL is responsible for transporting cholesterol from extrahepatic tissues to the liver through reverse cholesterol transport (Ebaid et al. 2020). This effect reinforces the role of melatonin in the integrated regulation of lipid metabolism and cholesterol homeostasis. Furthermore, studies show that melatonin reduces NF‐κB binding to DNA, which is important for the synthesis of enzymes that generate prostaglandins and reactive oxygen species (ROS), substances involved in inflammation (Hayden et al. 2006). NF‐κB is a regulator of cell survival, immunity, and inflammation and represents one of the pathways activated during hepatic injury and inflammation (He and Karin 2011). Thus, NF‐κB reduction leads to decreased production of proinflammatory cytokines and chemokines, explaining the reduced expression of IL‐6 and TNF‐α in the liver of melatonin‐treated animals (Chuang et al. 1996). Therefore, the anti‐inflammatory action of melatonin occurs through stimulation of Th2 lymphocyte proliferation, which produce IL‐4 and IL‐10, inhibiting the production of Th1 lymphocytes responsible for proinflammatory interleukin production.

However, studies indicate that melatonin also activates a secondary signaling factor (SIRT1), modulating STAT‐dependent signaling pathways, which contributes to repression of the inflammatory response and favors an anti‐inflammatory immunological profile characterized by increased IL‐4 and IL‐10 levels and reduced IL‐6 and TNF‐α expression. In this way, melatonin promotes an anti‐inflammatory environment in the liver, attenuating inflammation, lipotoxicity, and metabolic alterations associated with hyperlipidemia (Arioz et al. 2019; Chen et al. 2019).

## 5 Conclusions

The present study demonstrated that the administration of melatonin was shown to have protective effects on the factors of induction and development of nonalcoholic hepatic steatosis in Wistar rats with hyperlipidemia. This hormone can attenuate injuries and the development of liver disease by preventing damage caused by oxidative stress and reducing the binding of nuclear factor kappa B to the hepatocyte nucleus, thus reducing the production of proinflammatory cytokines. Consequently, it leads to reduced leukocyte infiltration, necrosis, and apoptosis, thus preventing morphological changes in cells and suppressing hepatic fibrosis. However, studies where melatonin is applied in the clinical treatment of liver damage and diseases are limited, being necessary for the future to conduct clinical trials to approve the use of this endolamine in this field.

## Author Contributions

Ana Cláudia Carvalho de Sousa and Jaiurte Gomes Martins da Silva conceived and designed the study, carried out the research, collected the data, and wrote the first draft of the manuscript. Érique Ricardo Alves, Laís Caroline da Silva Santos, Bruno José do Nascimento, Marcelle Mariana Sales de França, Vanessa Bischoff Medina, and Ismaela Maria Ferreira de Melo performed the research, collected the data, and assisted with laboratory analyses (biochemical analyses, histological processing, and immunohistochemistry). Álvaro Aguiar Coelho Teixeira and Valéria Wanderley Teixeira supervised the project, analyzed and interpreted the data, performed the statistical analysis, critically revised the manuscript, and acquired funding for the research. All authors contributed to and approved the final draft of the manuscript.

## Funding

This work was supported by Coordenação de Aperfeiçoamento de Pessoal de Nível Superior, 88882.436363/2019‐01.

## Conflicts of Interest

The authors declare no conflicts of interest.

## Data Availability

The data that support the findings of this study are available on request from the corresponding author. The data are not publicly available due to privacy or ethical restrictions.
